# Levan Exerts Health Benefit Effect through Alteration in Bifidobacteria Population

**DOI:** 10.29252/ibj.24.1.54

**Published:** 2019-08-29

**Authors:** Saeed Bahroudi, Bahareh Shabanpour, Joan Combie, Ali Shabani, Mona Salimi

**Affiliations:** 1 Department of Fisheries, Gorgan Agricultural Sciences and Natural Resources, Gorgan, Iran;; 2 Montana Biopolymers Inc., 119 Cathcart Circle, Winnsboro, SC 29180, USA;; 3 Physiology and Pharmacology Department, Pasteur Institute of Iran, Tehran, Iran

**Keywords:** Cholesterol, Levan, Prebiotics

## Abstract

**Background:**

Recently, exploring novel dietary nondigestible carbohydrates, which are able to influence the gut flora, has drawn much attention. The objective of this study was to find out the effective dose of levan, as a prebiotic, in rats in order to further apply in food industry.

**Methods:**

Levan at various doses (2-10%) was orally administered to male Wistar Albino rats once a day for 90 days. At the end of experiment, fecal and blood samples were collected to measure gut bacteria population and to carry out serum biochemical assay. The rats were sacrificed, and the colon tissues were stained with H&E and analyzed by histopathology.

**Results:**

Of note, levan effectively controlled body weight gain in the rats. Serum biochemical analysis revealed that 5% levan significantly diminished the serum level of total cholesterol, LDL, and glucose as well. More notably, 5% levan intake significantly increased the abundance of bifidobacteria population, highlighting its bifidogenic effect. Furthermore, our histopathological result revealed that daily intake of levan was associated with a higher degree of thickness of the mucosa layer compared to the rats in control group. Moreover, these findings manifested no colon inflammation in the rats fed with levan.

**Conclusion:**

The findings of this study provide the fundamental data to use levan at a definite dose for further development in functional foods.

## INTRODUCTION

A remarkable number of commensal bacteria present in the human gastrointestinal tract are in homeostasis with the host^[^^1^^]^. A symbiotic correlation, which is constructed with the host due to the presence of microbiota, has a great impact on health of the host by providing nutrients, causing conversion of metabolites, controlling epithelial cell proliferation, preventing growth of pathogens, and improving the immune system^[^^2^^]^. Hence, exploring the nondigestible food ingredients that cause specific changes in microbiota as well as the growth of the beneficial types of gut microbiota appears to be of great interest^[^^3^^]^. In this regard, prebiotics are emerging as the nondigestible food additives that stimulate the growth and activity of gut bacteria, including bifidobacterium, lactobacillus, and bacteroides, and consequently, improve the host health^[^^4^^]^. 

Fructan polysaccharides are one subtype of the prebiotics built up from fructosyl units, which are classified into two groups; levans and inulins, based on the type of fructose monomer linkage^[^^3^^]^. A growing number of studies have demonstrated the functionality of inulin; however, little is known about the biological function of levan^[^^5^^]^. The biological function of levan in a human diet begins with natto, a traditional Japanese food that people consume to promote long life and good health^[^^6^^]^. Levan is a fructosyl branched polysaccharide (β-2, 6) produced by a few plants and a wide range of both Gram-positive and -negative bacteria^[^^7^^]^.

According to the literature survey, levan has potential for use in food, pharmaceutical, medicine, cosmetic and industrial sectors or can be exploited in a drug delivery system^[^^6^^]^. Multiple pharmacological as well as functional properties, including anti-tumor, cholesterol-lowering, anti-atherosclerotic, anti-pathogenic, anti-irritant, anti-oxidant, and anti-inflammatory effects have been attributed to levan^[^^8^^]^. Noteworthy is that some *in vitro* studies have shown the potential modulatory effect of levan on colon microbiota^[^^9^^,^^10^^]^, but few researchers have studied its *in vivo *efficacy^[^^11^^-^^13^^]^. Thus, in the present study, we attempted to determine the efficacy of oral administration of levan at different doses in rats by assessing the major gut flora populations and thickness of colon tissue, as well as by measuring the biochemical parameters of blood.

## MATERIALS AND METHODS

**Animals and treatment**

Male Albino Wistar rats (4 weeks old, 100 ± 20 g) were obtained from the National Animal Center (Pasteur Institute of Iran, Karaj) and maintained in a 12/12 h light-dark cycle, with food and water supplied *ad libitum*. The body weight was measured once a week. Animals were treated in accordance with the guidelines approved by the animal Ethics Committee of Pasteur Institute of Iran (IR.PII.REC.1397.027). Levan was kindly gifted by Montana Biopolymers Company (USA) and solubilized in drinking water to obtain a range of doses between 2-10% (W/V). Thereafter, 1 ml of the prepared solutions were orally administered to the rats, which were randomly divided into six groups (n = 5): 1-4, that were fed daily by 2%, 5%, 7%, and 10% levan solutions, respectively; 5, positive control group was fed by 5% inulin solution;6, negative control group was gavaged with water. 

**Blood sample collection and biochemical analysis**

Following a 90-day feeding period, rats (n = 3) were anesthetized, and then the blood was collected by heart puncture into heparinized tubes. After centrifugation at 2500 ×g for 15 minutes, the blood serum samples were kept at -80 °C for biochemical analysis. Total cholesterol, triglyceride, LDL, HDL, and glucose concentrations in serum were determined using commercial cholesterol (CHOD), triglycerides (GPO-PAP), LDL-C, HDL-C (Photometric), and glucose (GOD) kits (Pars Azmun Co., Alborz, Iran), respectively, based on the manufacturer’s instruction.

**DNA extraction**

DNA was extracted from fecal samples. To obtain fresh fecal samples at the end of experiment, the rectal region was gently squeezed, and then the rats were sacrificed. Total DNA extraction and purification were carried out using QIAamp DNA Stool Mini Kit (Qiagen, USA) according to the manufacture’s instruction. DNA concentration was measured spectrophotometrically by using a picodrop spectrophotometer (Picopet 01, Picodrop Ltd., Cambridge, UK). Purified DNA samples were stored at -80 °C.

**qPCR analysis**

The qPCR was used to evaluate the effect of levan at different doses on microbial composition in colon of rats after 90 days of treatment in comparison with the control. Various microbial groups, including bacteroides, lactobacilli, and bifidobacteria were identified and quantified using qPCR. The 16S rRNA-generated group-specific primers used in this study are listed in Table 1, and qPCR assays were carried out based on the method described by Rinttilä *et al.*^[^^14^^]^ using a Rotor-Gene 6000 instrument (Corbett Life Science, Australia).

**Table 1:** Specific 16S rRNA primers sequences used for qPCR

Target bacterial group	Primer	Sequence (5′−3′)	PCR product size (bp)	Ref.
**Bifidobacteria**	F-Bifido	CGCGTCYGGTGTGAAAG	244	^[^^23^^]^
	R-Bifido	CCCCACATCCAGCATCCA		
				
**Lactobacilli**	F-Lacto	GAGGCAGCAGTAGGGAATCTTC	126	^[^^23^^]^
	R-Lacto	GGCCAGTTACTACCTCTATCCTTCTTC		
				
**Bacteroides**	F-AllBac 296	GAGAGGAAGGTCCCCCAC	106	^[^^24^^]^
	R-AllBac 412	CGCTACTTGGCTGGTTCAG		

**Colon histology and determination of intestinal mucosa layer thickness**

Following washing the large intestine in physiological saline (0.9% NaCl), the tissues were fixed in 4% neutral formaldehyde solution for 48–72 h. The residual fixative was removed, and then the samples were washed, dehydrated and embedded in paraffin blocks using classical procedures. After staining with H&E, the samples were observed by light microscopy at magnification 100×.

**Statistical analysis**

All the results were analyzed using GraphPad Prism version 6. One-way analysis of variance (ANOVA) followed by post test Tukey’s was used to determine the difference among treatments. In all analyses, a *p *< 0.05 was considered statistically significant. Data were expressed as mean ± SEM in triplicate.

## RESULTS

**Weight gain of the rats**

All groups were provided with different treatments of prebiotics, inulin and levan at different doses. As results showed, on the first day of experiments, the mean body weight of the rats was 105.25 ± 14.39 g and at the end of the experimental period, it went up to 300.20 ± 24.29 g in the control group. At the end of the experiments in the treated groups, the mean body weight increased by 175.21% in inulin, 132.14% in levan 2%, 114.71% in levan 5%, 125.22% in levan 7%, and 131.81% in levan 10% groups. The group subjected to 5% levan exhibited a better control of weight gain after 12 weeks of administration, as compared to the other groups. 

**Effect of levan on biochemical parameters**

After 12 weeks of treatment, levels of serum glucose, total triglyceride, cholesterol, LDL, and HDL were measured, and the results are shown in Table 2. Among all the treated groups, a significantly decreased LDL level was detected in the rats fed with levan 5%, whereas no significant reduction was found in LDL level following treatment with inulin and levan 2%. Similarly, total concentration of triglyceride and cholesterol diminished in the group treated with levan 5%. Surprisingly, higher doses of levan (7 and 10%) elevated the level of LDL in the serum of treated rats. Noteworthy is that levan 5% was more effective than inulin in lowering triglyceride, cholesterol, and LDL levels. The HDL level also remained unchanged in all the treated groups. Additionally, the serum glucose of rats fed with levan 5% was significantly lower than that of the control rats (*p* < 0.05). However, the serum level of glucose rose in the rats fed with the doses higher than levan 5% (Table 2), which may be attributed to the level of D-glucose present in the levan (43%). 

**Effect of levan on fecal microbiota content**

**Fig. 1:**
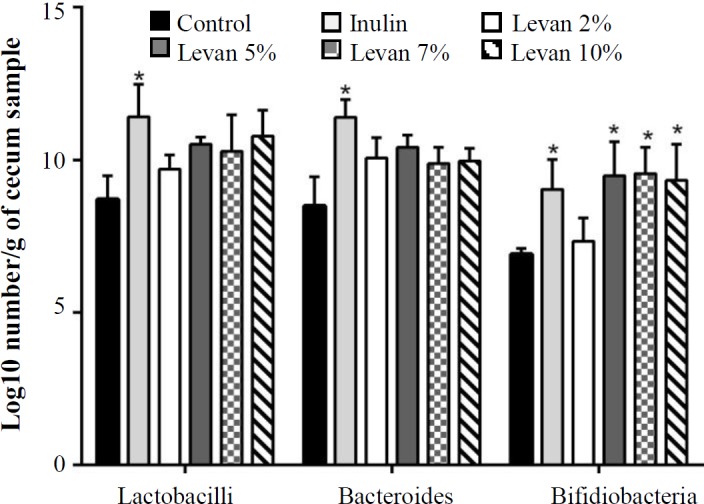
Effect of different doses of levan on cecal microbiota populations in rats fed for 90 days. Bars marked with asterisks are significantly different compared with the control group (*p* < 0.05). Inulin was used as the positive control group

Quantitative differences between bacterial groups in the fecal samples of rats fed with control, inulin 5%, and different doses of levan were evaluated by using qPCR. Following a period of 90 days of treatment, the number of bifidobacteria was significantly greater in rats fed with levan 5, 7, and 10% (*p* < 0.05) compared with the control group, whereas no significant increase was noted in the number of lactobacilli and bacteroides between the control group and the groups treated with different doses of levan (Fig. 1). Although lactobacilli and bacteroides populations were unaffected by levan, inulin, as a positive control, exerted a significant effect on these populations. Levan appeared to stimulate Bifidobacterium spp. growth in the gut.

**Improvement in intestinal mucosa layer thickness**

**Fig. 2:**
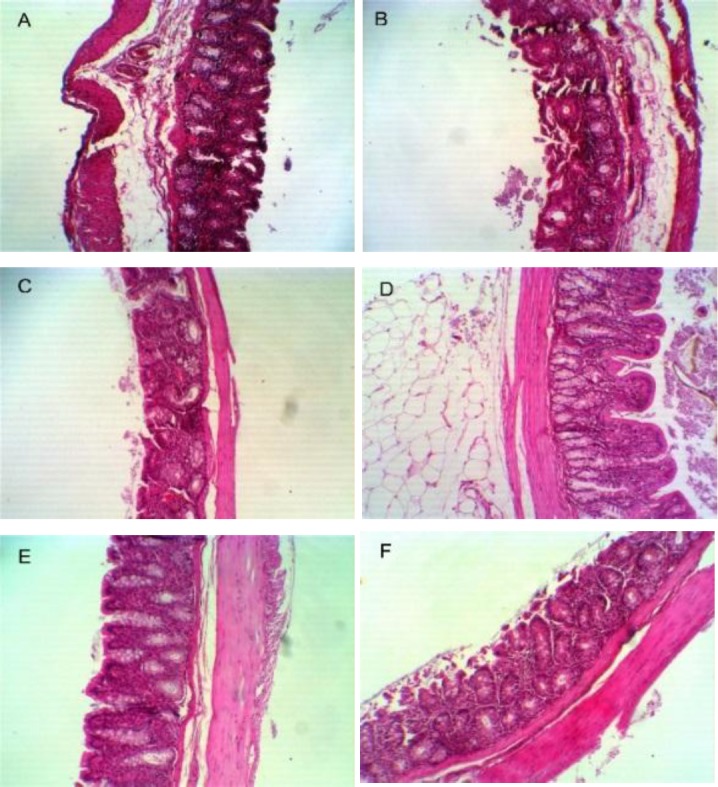
Analysis of colon sections of rats and mucosa layer thickness in control (A), inulin (B), levan 2% (C), levan 5% (D), levan 7% (E), and levan 10% (F).

In order to evaluate the effect of levan on the thickness of the colonic mucus layer, colon tissues were dissected and stained by H&E. Our results showed that the thickness of mucosal layer increased from 30 µm in the control group to 45, 35, 55, 40, and 50 ± 5 µm in inulin and the groups treated with levan 2%, 5%, 7%, and 10%. This study demonstrated that levan 5% caused a considerable increase in the thickness of the rat colon mucosal layer, which indicates an elevated cellular proliferation supplying a protective function to the gut. Integrity of the mucosal tissue containing intestinal glands was improved in rats receiving levan, particularly levan 5% (Fig. 2). No histological abnormalities were found in the colonic mucosa of animals from all the groups. 

**Table 2:** Glucose, triglyceride, total cholesterol, LDL, and HDL levels in serum in control and experimental groups

Groups	Glucose	Triglyceride	Total cholesterol	LDL	HDL
**Control**	92.00 ± 4.61^a^	99.00 ± 10.39^a^	66.50 ± 2.50^a^	19.83 ± 2.07^a^	23.80 ± 1.67^a^
**Inulin**	71.33 ± 4.84^a^	97.00 ± 0.57^a^	70.33 ± 0.33^a^	14.35 ± 3.35^a^	25.00 ± 0.60^a^
**Levan 2%**	62.25 ± 8.25^a^	94.25 ± 13.25^a^	62.00 ± 2.30^a^	13.85 ± 3.65^a^	22.23 ± 1.23^a^
**Levan 5%**	49.00 ± 1.73^b^	93.00 ± 3.00^a^	45.33 ± 2.60^b^	5.70 ± 0.60^b^	19.95 ± 0.75^a^
**Levan 7%**	59.50 ± 2.50^b^	103.50 ± 5.50^a^	65.67 ± 1.20^a^	23.20 ± 2.06^a^	20.53 ± 1.12^a^
**Levan 10%**	100.8 ± 14.25^a^	100.00 ± 1.00^a^	70.00 ± 1.73^a^	30.13 ± 1.01^a^	22.60 ± 1.40^a^

Data represent mean ± standard error. Different superscript letters indicate a significant difference (*p* < 0.05) compared with the control.

## DISCUSSION

Dietary fiber is accepted worldwide for its benefits on health and well-being, including effects on bowel habit, fermentation in colon, reduction of blood (LDL-) cholesterol level, and improvement of blood glucose and insulin levels^[^^3^^]^. In the present work, we assessed the effects of different doses of levan, as a prebiotic, on a part of colon microbiota populations and then evaluated its health effect on the rat, as an animal model. 

Accumulating evidence indicates that a diet containing high fiber is critical for preventing overweight by the satiating effect. In this regard, some prebiotic fibers have been shown to contribute to the enhanced feeling of satiety and subsequent less energy intake^[^^15^^]^. Consistent with these findings, our results demonstrated that daily intake of levan led to a decreased weight gain in rats in a dose-dependent manner. Our results are in line with the study performed by Belghith *et al.*^[^^11^^]^, supporting the role of levan as a prebiotic fiber in weight control.

In this study, we observed cholesterol-, LDL-, and glucose-lowering effects, but not triglyceride and HDL-lowering impacts in rats fed with 5% levan. In support of our results, Yamamoto *et al.*^[^^13^^]^ found a similar hypocholesterolemic effect of levan. Interestingly, rats fed with levan at doses more than 5% showed no remarkable hypolipidemic and hypoglycemic effects compared with the control group. The discrepancy in the effects of high doses of levan may be due to the free monosaccharaides including glucose and fructose present in the levan as impurities. This supposition was further confirmed by determining the D-glucose and D-fructose contents in the provided levan. Our assay indicated that around 50% of levan contains free monosaccharaides. It is notable mentioning that despite the presence of monosaccharaides in levan, it could effectively work as hypolipidemic agent at the dose of 5%. Given our results, we showed a greater efficacy of levan compared with inulin as a positive control to beneficially modulate blood biochemical parameters, specifically the LDL level.

Levan at different doses could not significantly elevate the lactobacilli and bacteroides populations in faeces of rats compared with the control group, which may be due to inadequate intake of levan solution to modulate these types of gut microbiota. Consistent with these results, previous studies have found a broad range of levan effects on different types of gut microbiota^[^^9^^,^^16^^,^^17^^]^. It is remarkable that in our study the bifidobacterial population raised following levan administration at doses higher than 2% for 90-day treatment periods. However, no significant difference was seen in the levels of bifidiobacterial population in the fecal samples of rats fed with 5, 7, and 10% of levan. Notably, the bifidogenic effect of levan was observed at a moderate dose (5%), which could be beneficial to avoid possible side effects including intestinal discomfort due to gas production^[^^18^^]^. Interestingly, the bifidogenic effect of levan was similar to 5% of inulin as a positive control. Our results are promising since bifidobacteria generate antibacterial agents and/or inhibit the adhesion of pathogens via acetic acid production, which in turn exerts a protective role against pathogens^[^^18^^,^^19^^]^. Keeping in mind this point and knowing that levan can act as an anti-pathogenic substance^[^^20^^]^, the intake of levan, as a prebiotic, may contribute to the well-being of the host. 

It has recently been accepted that the mucus barrier plays a decisive role in regulating interaction with the intestinal microbiota. It has also been found that the mucus layer serves as a niche for microbiota to digest complex glycoproteins in order to use as a carbon source. Moreover, gut microbial community is associated with mucosa layer thickness. In this regard, previous studies have shown that increase in the population of gut micobiota and subsequent related metabolites leads to the increased thickness of the mucosa layer^[^^3^^,^^21^^]^. In accordance with these data, our findings indicated that upon feeding the rats with levan 5% or more, the mucosa layer thickness increased. This observation suggests that short chain fatty acid productions by gut bacteria can contribute to the thickness of the mucosal layer, which is in line with previous reports ^[^^3^^,^^22^^]^. Importantly, the impact of levan doses was slightly more than that of the rats fed with inulin as positive control, demonstrating the potential prebiotic functionality of levan when used as a food ingredient.

In summary, using an animal model in this study, we clearly demonstrated a bifidogenic effect for 5% levan as well as a considerable potential to reduce LDL, cholesterol, and glucose levels. Furthermore, findings of the present study suggest that the inclusion of this prebiotic in diet assists in controlling weight gain. Overall, levan may be a useful candidate, as an ingredient, in functional food products for any long-term effect. 
